# Translating digital health services for nutrition care management of chronic conditions in outpatient settings: A multi‐stakeholder e‐Delphi study

**DOI:** 10.1111/1747-0080.12927

**Published:** 2025-02-17

**Authors:** Amandine Barnett, Ingrid J. Hickman, Katrina L. Campbell, Jaimon T. Kelly

**Affiliations:** ^1^ Centre for Online Health The University of Queensland Brisbane Queensland Australia; ^2^ Centre for Health Services Research The University of Queensland Brisbane Queensland Australia; ^3^ Faculty of Medicine The University of Queensland Brisbane Queensland Australia; ^4^ ULTRA Team The University of Queensland Clinical Trials Capability Brisbane Australia; ^5^ Healthcare Excellence and Innovation, Metro North Health Brisbane Queensland Australia

**Keywords:** chronic disease, consensus study, dietetics, digital health, implementation, telehealth

## Abstract

**Aim:**

To identify and achieve expert consensus on the most important and feasible strategies to implement digital health services for nutrition care management of chronic conditions in outpatient settings.

**Methods:**

Determinants and strategies for implementing digital health services for nutrition care management were identified in line with the literature and the Consolidated Framework for Implementation Research. These were presented to team leaders and senior clinicians, as well as dietetic and allied health directors in a two‐round e‐Delphi process. Consensus was reached when strategies were rated very important/feasible by at least 75% of respondents, calculated by the median, interquartile range and frequency. Following the two survey rounds, a final prioritisation survey was distributed to participants, where participants were asked to prioritise their top strategy for each determinant, which was analysed by frequency calculations.

**Results:**

Twenty participants participated in round one of the survey and 18 completed the final prioritisation round. Following the two rounds, 3 strategies did not meet consensus for importance and 7 strategies did not meet consensus for feasibility out of 25 strategies presented. Nine strategies were prioritised following the survey rounds. Key concepts of the strategies that met consensus and were prioritised related to (i) adhering to quality of care with effective evaluation processes; (ii) providing options for digital health upskilling and support; and (iii) individualising patient care.

**Conclusion:**

Team leaders and senior clinicians as well as dietetic and allied health directors have indicated that there are many important digital health strategies yet not all are feasible to implement within current resourcing and systems.

## 1 INTRODUCTION

Chronic conditions place a significant burden on our health system, lead to premature death and consume healthcare resources.^1^ Each year, ~41 million individuals lose their lives to non‐communicable chronic conditions worldwide.^2^ Poor dietary behaviours can contribute to this^2^ for which specialised nutrition therapy could be beneficial. Delivering nutrition therapy at the scale required to meet the needs of growing chronic conditions is challenging^3^, ^4^ and could benefit from innovative models of care. Digital nutrition services are one of these models; however, understanding how these models of care can be feasibly incorporated into existing health systems has not been evaluated.

Digital health relates to the knowledge and practice of digital technologies to improve health and includes the utilisation of a wide range of smart devices and equipment which connect to the internet.^5^ This includes telehealth (i.e., audio and video consultations). The use of telehealth and digital health is increasing exponentially and so is the research on its effectiveness and acceptability. This was accelerated during the COVID‐19 pandemic and following the permanent telehealth item number introduction into the Australian Medicare system in 2022.^6^ At a patient level, it is perceived to overcome travel burden associated with attending appointments, improve self‐management by being more actively involved in their healthcare and it may facilitate faster communication with clinicians allowing treatment to be delivered more promptly.^7^ While at the health system organisation level, digital health has been shown to reduce hospitalisation rates,^8^ reduce waste in resources^9^ and improve automation of tasks by up to 30%.^4^ In a nutrition specific context, these tools have shown to modestly improve eating behaviours in individuals with chronic conditions.^10^, ^11^ Although digital health can deliver a range of benefits, there remains opportunities to strengthen the implementation of these services, including those to support nutrition care.

Strategic planning is needed to overcome barriers related to organisational processes of digital health. Dietitians, doctors, nurses, other allied health clinicians and health directors (going forward, we will refer to this groups as ‘healthcare providers’) have consistently expressed that certain determinants (barriers and facilitators) can influence adoption of digital health with a few indicating strategies to address these determinants.^12^, ^13^, ^14^, ^15^, ^16^ Considerations for a wide range of literature to assist with identifying strategies is needed due to the limited availability of research within a nutrition‐specific context, with only one known study that focuses on organisational determinants of digital nutrition health.^17^

Furthermore, continued adoption of digital health in hospital settings means evolving staff roles, infrastructure and policy changes,^4^ thereby influencing the relevance of the strategies drawn from past research. A major global challenge relates to resourcing availability, allocation and constraints,^5^ thus assessing the feasibility of these strategies based on existing resource arrangements is also an important consideration. Therefore, it is timely to put the latest evidence on digital health implementation to key stakeholders to establish consensus on the most important and feasible strategies to implement digital health services for nutrition care management of chronic conditions in outpatient settings.

## 2 METHODS

A modified e‐Delphi process was utilised in this study. The Delphi process has been widely used in health service research for gathering opinions and consensus from a stakeholder panel.^18^ This methodology involves stakeholders who can offer expert opinion and typically have the power to implement decisions.^19^ It allows experts to consider the complexity of the problem and share specific attitudes towards the acceptance of a number of strategies for varying determinants.^19^ The study complied with the guidelines for Conducting and REporting Delphi Studies (CREDES).^20^ Ethical approval was granted from the Human Research Ethics Committees for Metro South Hospital and Health Service (HREC/2023/QMS/94426) and ratified by The University of Queensland (2023/HE001282).

**FIGURE 1:**
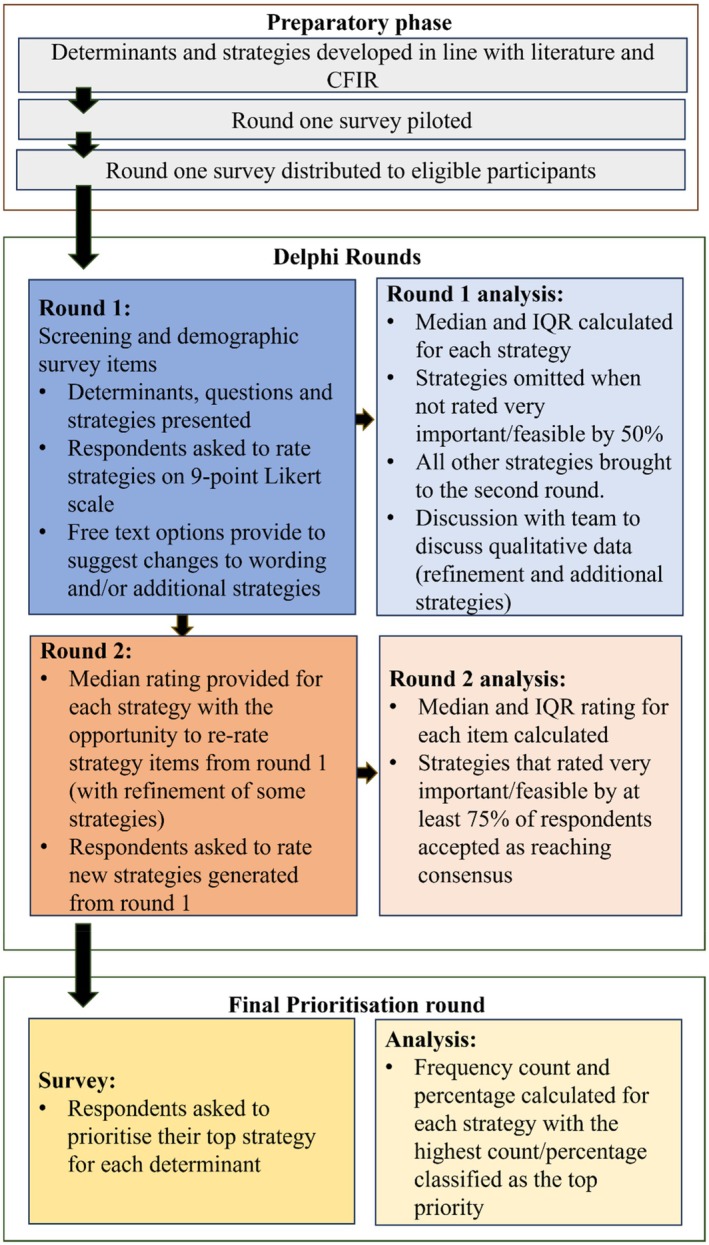
A diagram of the e‐Delphi procedure. CFIR, Consolidated Framework of Implementation Research; IQR, interquartile range.

For this study, the e‐Delphi process involved two rounds where a diverse panel of experts were presented with determinants, questions, and strategy statements. In the first round of the survey, participants were asked to rate strategy statements and had the opportunity to provide feedback on presented material. In the second round of the e‐Delphi survey, participants were presented with the anonymous responses from the previous round and asked to re‐rate strategies to determine consensus, including any additional questions suggested by participants from round one. We modified the e‐Delphi process to include a prioritisation survey for the third and final round, where participants were asked to choose one strategy for each determinant that could be prioritised for implementation. An overview of the e‐Delphi procedure is presented in Figure 1.

The expert panel was recruited using purposive and snowballing sampling methods. Two groups of healthcare providers working within Metro South Hospital and Health Services in Brisbane, Australia (inpatient and outpatient hospital settings), were invited to participate in the study between August 2023 and November 2023. These included (1) directors of dietetics and allied health; (2) team leaders and senior clinicians (dietitians, nurses, registrars, consultants, or other allied health clinicians) who are involved in making decisions for the design of services. Clinicians could self‐identify as being in a senior role. Participants were initially sought through contact via dietetic and allied health directors who were asked to distribute the study information to senior clinicians within their department and requests for the lead researcher to discuss the study at one of their department meetings. Email contact through the research team networks and snowballing approaches were adopted to recruit senior nurses, registrars, consultants, and other allied health clinicians. Implied informed consent was determined by the commencement of the survey. The minimum target sample was 20–25 participants, where a 20% drop out was considered for each round, to adhere to recommendations for minimum samples for Delphi studies.^21^, ^22^ All participants who completed the first round of the survey were invited to complete Round 2 and the final prioritisation survey.

The initial determinants (barriers and facilitators) and strategies for the survey were informed by a targeted literature search on digital health, healthcare providers' perspectives,^12^, ^13^, ^14^, ^15^, ^16^, ^17^ and chronic disease management,^23^, ^24^, ^25^, ^26^, ^27^ with a focus on implementation barriers and facilitators. This literature search was conducted across key biomedical electronic databases to identify these investigations. The determinants and strategies were then mapped to the domains of the consolidated framework for implementation research (CFIR), mainly the inner setting and implementation process domains.^28^ Determinants related to wider barriers or facilitators, which the specific strategy relates. The determinants that were included in this study were (a) administration support; (b) value, norms and beliefs of healthcare providers towards digital health; (c) perceived consumer demand; (d) adherence to quality care; (e) staff training; (f) patient support; (g) sharing of resources and learnings; (h) individualising dietary information. Definitions of digital health and feasibility in the context of this study were provided to participants. Digital health services referred to video conferencing, mobile applications, text messaging programs, wearable devices and web/computer‐based programs. Feasibility was defined as the extent to which a strategy could be easily implemented by a director or senior clinician within the current resourcing at their department.

Data collection occurred from August 2023 to May 2024. The survey was administered via the Qualtrics survey platform.^29^ Participants were provided with a link to complete the survey via email. Participants were asked to complete each round of the e‐Delphi within 3 weeks of being sent the survey and were sent email reminders (weekly then everyday 2 days when overdue and reminders were stopped if there was nil response after the second attempt). Dietitians were involved in the piloting of the round one survey to check readability and ensure that the survey platform was functioning well. The research team discussed any potential refinement of the survey as per feedback from the piloting phase which included formatting and restructuring of the questions. The mapped determinants and strategies used in the e‐Delphi can be found in Table S1.

Participants were screened and asked to provide information about their demographics (age, gender, discipline, experience in current role and experience with digital health). Once these initial questions were completed, participants had access to the main survey. The survey was structured by firstly presenting a determinant followed by a related question alongside two to four strategies related to the determinant in consideration. There were eight determinants and 23 strategies that were initially presented in round one. Participants were invited to rate each of the strategies separately according to their (a) importance and (b) feasibility on a nine‐point Likert Scale. For each set of strategies, participants had the opportunity to make suggestions to the wording of the strategies presented via a free text option and/or suggest their own additional strategies. All responses collected were anonymised to ensure confidentiality. Additional strategies and changes were decided upon discussion with the research team before presenting to the participants in the subsequent rounds. Qualitative content analysis^30^ was used to analyse the free text data.

Descriptive statistics were performed using SPSS statistical software (IBM Version 29) to analyse participants' demographics, rated and prioritised strategy items. Median and interquartile range are the more robust measures of presenting the consensus.^31^ The median and interquartile range rating for each strategy were calculated and recoded as: ‘1–3’ not very important/feasible; ‘4–6’ moderately important/feasible; ‘7–9’ very important/feasible. Frequency calculations were used to determine agreement within the group. Strategies that were rated <50% for ‘very important’ or ‘very feasible’ were omitted in round one and the rest, which scored 50% or higher, were brought into the second round of the survey.^18^

Participants were asked to consider their round one response in reference to the broader group's aggregated results and re‐rate the strategies. Participants were also asked to rate the new strategies suggested by participants from round 1. Final consensus was calculated for each of the strategies, at the end of round 2. Consensus was achieved when there were 75% or more agreement by participants who rated a strategy as very important/feasible.^32^ The median and interquartile range rating for each strategy were calculated and recoded using the same criteria as round one.

Participants were asked to prioritise their top strategy for each determinant in a final prioritisation survey. The frequency count and percentage were calculated for each strategy to determine the top prioritised strategy with each determinant domain.

## 3 RESULTS

Twenty participants completed at least one main survey question in round 1. All participants worked in a hospital setting, with one also working in a community health centre. Five senior clinicians and team leaders indicated they worked across multiple chronic disease areas of practice. Most participants (*n* = 17/20) had experience with using both video consultations (telehealth) and electronic health (i.e., web‐based resources, emailed resources) while only half of the participants reported that they had experience with mobile health services (i.e., mobile applications via smartphones or tablets, wearable devices) (*n* = 10/20). Participants characteristics are summarised in Table 1.

**TABLE 1:** Participant demographics for round 1 (*n* = 20).

Variable	Number (%)
Age	
25–40 years	5 (25)
41–50 years	7 (35)
51–60 years	7 (35)
61–70 years	1 (5)
Gender	
Female	17 (85)
Male	3 (15)
Stakeholder group	
Dietetic or allied health director	3 (15)
Team leader	3 (15)
Senior clinician	14 (70)
Discipline	
Dietetics	10 (50)
Other allied health	1 (5)
Medicine	6 (30)
Nursing	3 (15)
Area of practice (senior clinicians and team leaders)^a^	
Diabetes	5 (25)
Obesity	3 (15)
Cardiovascular	1 (5)
Gastroenterology	4 (20)
Renal	10 (50)
Other	2 (10)
Years of experience	
1–3 years	2 (10)
4–6 years	1 (5)
7–9 years	4 (20)
10–20 years	9 (45)
>20 years	4 (20)

^a^ Participants could indicate multiple areas of practice.

Three strategies were not rated important/feasible by at least 50% of participants (one for importance and two for feasibility) and were dropped from the round 2 survey. As a result of the group feedback from round one, some (*n* = 4) strategies were refined and additional strategies (*n* = 3 for importance and *n* = 3 for feasibility) were included, which were then presented to participants for rating in round 2. The total number of strategies at the end of round 1 analysis was 25 strategies (noting that one strategy was presented only for importance but not feasibility). Results from round 1 also indicated that a clear definition of feasibility and importance needed to be included in the second round of the e‐Delphi survey.

There were 16 out of 20 participants who completed round 2 of the e‐Delphi survey (80% response rate). Out of 25 strategies, 3 strategies did not meet consensus for importance and 7 strategies did not meet consensus for feasibility. Out of 25 strategies, 17 (68%) that met consensus were rated higher for importance than feasibility. A summary of results for rounds 1 and 2 are provided in Table 2.

**TABLE 2:** Summary of rounds 1 and 2 results.

	Importance						Feasibility					
	Round 1 (*n* = 20)			Round 2 (*n* = 16)			Round 1 (*n* = 20)			Round 2 (*n* = 16)		
Determinant category (bolded) and strategies (listed below)	*M*	IQR	% rating very important	*M*	IQR	% rating very important	*M*	IQR	% rating very feasible	*M*	IQR	% rating very feasible
*Administration support*												
Involve allied health assistants to assist with administrative tasks related to digital health	6.0	4.0–7.8	35.0				5.0	3.0–7.0	40.0			
Develop a business case to support the creation of new digital health administrator roles	8.0	5.3–9.0	65.0	8.0	6.3–8.0	75^b^	6.5	4.3–7.0	50.0	7.0	6.0–7.8	56.5
Investigate how the booking systems can be adapted to better support digital health services.	8.0	7.0–9.0	90.0	8.0	7.0–8.8	93.8^b^	7.0	6.0–9.0	60.0	7.0	6.0–8.0	62.5
A clearer understanding and outline of the different organisation and staffing roles related to digital health	7.0	7.0–8.3	80.0	7.5	6.3–9.0	75^b^	7.0	6.3–8.0	75.0	7.0	6.3–7.8	75.0^b^
Provide training to administrative officers to perform a digital health administrative role^a^				8.0	7.0–9.0	81.3^b^				7.5	7.0–8.8	75.0^b^
*Value, norms and beliefs*												
Promote telehealth to staff as a hybrid model that can enhance consumer‐centred care	8.0	7.0–9.0	78.9	8.0	6.0–9.0	68.8	8.0	7.0–9.0	84.2	8.0	7.0–9.0	93.8^b^
Designate digital health champions who would be responsible for promoting digital health services in their departments/wards	7.0	6.0–9.0	68.4	7.0	6.0–8.8	56.3	7.0	7.0–9.0	78.9	7.0	7.0–8.0	81.3^b^
Share past digital health projects and positive experiences – sharing success using the inter‐site and interdisciplinary meetings	7.0	5.0–9.0	57.9	7.0	6.3–8.0	75^b^	8.0	7.0–9.0	78.9	8.0	7.0–9.0	81.3^b^
Encouraging healthcare providers (particularly team leaders and directors) to be involved in digital nutrition research projects.	7.0	6.0–9.0	63.2	7.0	6.3–8.0	75^b^	7.0	6.0–9.0	68.4	7.0	6.0–8.8	62.5
*Perceived consumer demand*												
Promote telehealth to staff as a hybrid model that is evidence‐based and enhances consumer‐centred care.	9.0	7.0–9.0	89.5	8.5	7.3–9.0	81.3^b^	8.0	7.0–9.0	89.5	8.0	7.0–8.8	81.3^b^
Set and evaluate KPIs for digital nutrition services which are realistic for the healthcare provider, considers the consumer demand and consumer suitability for the modality of contact	8.0	5.0–9.0	68.4	8.0	7.0–8.8	68.8	8.0	6.0–9.0	73.7	7.5	6.0–8.0	68.8
Educating healthcare providers of the perceived acceptability of digital health services across all groups^a^				7.5	6.0–8.0	87.5^b^				7.0	7.0–8.0	81.3^b^
*Adherence to quality of care*												
Audit and evaluate digital health services including consumer reported outcome and experience measures.	9.0	7.0–9.0	94.7	9.0	8.0–9.0	93.8^b^	8.0	7.0–9.0	78.9	8.0	7.0–8.8	87.5^b^
Establish ongoing quality improvement projects.	9.0	7.0–9.0	94.7	9.0	8.0–9.0	93.8^b^	7.0	6.0–9.0	73.7	7.5	7.0–8.8	81.3^b^
Mandate reporting and mitigation of adverse events that occur during telehealth appointments.	9.0	7.0–9.0	94.7	8.0	8.0–9.0	100.0^b^	7.0	6.0–9.0	68.4	8.0	7.0–8.0	81.3^b^
*Staff training*												
Conduct discipline‐specific assessments, rapport building and online communication, simulated practice sessions.	7.0	6.0–9.0	73.7	7.0	7.0–8.0	93.8^b^	7.0	6.0–8.0	73.7	7.0	6.0–8.0	68.8
Provide staff delivering services with ‘how to’ guides and troubleshooting guides.	8.0	7.0–9.0	84.2	8.0	6.3–9.0	75.0^b^	8.0	7.0–9.0	78.9	8.0	8.0–8.8	100.0^b^
Provide staff the option to participate in training related to digital health services.	7.0	6.0–9.0	68.4	8.0	7.0–8.0	100.0^b^	7.0	5.0–9.0	63.2	7.0	7.0–8.0	87.5^b^
*Patient support*												
Provide consumers with ‘how to’ guides and troubleshooting guides.	9.0	7.0–9.0	89.5	9.0	8.0–9.0	100.0^b^	8.0	7.0–9.0	78.9	8.0	8.0–9.0	93.8^b^
Have onboarding and technical support available for those who need and make this available over the weekend and after work hours.	9.0	9.0–9.0	94.7	8.5	7.3–9.0	87.5^b^	7.0	5.0–8.0	94.7	6.0	5.0–7.0	43.8
Develop a business case for consumers to receive after‐hours technical support^a^				8.0	6.3–8.8	75.0^b^				6.0	4.0–8.0	50.0
*Sharing of resources and learnings*												
Access to a library of digital resources to enable consolidation of resources across disciplines, services, and professional bodies	8.0	7.0–9.0	84.2	8.0	7.3–9.0	93.8^b^	8.0	7.0–9.0	84.2	8.0	7.0–8.8	81.3^b^
Use existing meetings to monitor implementation of digital health interventions.	7.0	7.0–9.0	84.2	8.0	7.0–8.8	87.5^b^	7.0	6.0–9.0	68.4	8.0	7.0–8.0	81.3^b^
*Individualising dietary information*												
Continue to provide ongoing appointments (in‐person or video telehealth) with dietitians, to those who request	8.0	8.0–9.0	84.2	9.0	8.0–9.0	100.0^b^	7.0	7.0–9.0	78.9	8.0	8.0–9.0	100.0^b^
Dietitians need to individualise the material that is shared to patients through digital health.	8.0	7.0–9.0	78.9	9.0	8.0–9.0	100.0^b^	7.0	6.0–8.0	63.2	8.0	7.0–8.8	87.5^b^
Allow for mobile and web‐based services to have a feature where it is customisable, and the settings can be changed to suit the patient/users, e.g., number of push notifications received.	9.0	7.0–9.0	94.7	9.0	8.0–9.0	100.0^b^	6.0	5.0–7.0	47.4			

*Note*: Grey shading indicates a strategy that was not asked during a survey round.

Abbreviation: M = median, KPI= key performance Indicators.

^a^ Additional strategies as per group feedback.

^b^ A strategy that reached consensus.

There were 18 participants who completed the final prioritisation round of the survey (90% response rate). Nine strategies were prioritised by the group for the eight determinants that were presented (Table 3). Two strategies, *adherence to quality of care* and *sharing of resources and learnings*, had the highest frequency calculation (*n* = 14/18, 77.8%). The frequency calculation for the other strategies prioritised was below 56%. The total frequency count and percentages for each strategy can be found in Table S2.

**TABLE 3:** Top strategy prioritised for each determinant evaluated, *n* = 18.

Determinant category	Strategy	Frequency (%)
Adherence to quality care	Audit and evaluate digital health services including consumer reported outcome and experience measures.	14 (77.8)
Sharing of resources and learnings	Access to a library of digital resources to enable consolidation of resources across disciplines, services, and professional bodies.	14 (77.8)
Value, norms and beliefs	Promote telehealth to staff as a hybrid model that can enhance consumer‐centred care.^a^	10 (55.6)
Patient support	Provide consumers with ‘how to’ guides and troubleshooting guides.	8 (44.4)
Administration support	Provide training to administrative officers to perform a digital health administrative role.	8 (44.4)
Individualising dietary information	Continue to provide ongoing appointments (in‐person or video telehealth) with dietitians, to those who request. AND Allow for mobile and web‐based services to have a feature where it is customisable, and the settings can be changed to suit the patient/users, e.g., number of push notifications received.	8 (44.4)
Perceived consumer demand	Promote telehealth to staff as a hybrid model that is evidence‐based and enhances consumer‐centred care.	7 (38.9)
Staff training	Provide staff delivering services with ‘how to’ guides and troubleshooting guides.	7 (38.9)

^a^ A strategy that was prioritised and met consensus for feasibility but not importance.

## 4 DISCUSSION

This e‐Delphi study aimed to identify and build consensus with healthcare providers on the most important and feasible strategies to implement digital health services for nutrition care management of chronic conditions in outpatient settings. A high number of strategies presented in this study met consensus for importance and/or feasibility. Out of 25 strategies, 17 that met consensus were rated higher for importance than feasibility. Strategies that met consensus for both importance and feasibility related to broader determinants of adhering to quality of care with effective evaluation processes, providing options for digital health upskilling and support and individualising patient care. These determinants were further reflected in the strategies that were prioritised in the study's final survey.

There were more strategies that met consensus for importance than for feasibility, indicating that digital nutrition models may be harder to implement for healthcare providers despite being highly important. This could be related to participants' insufficient resources and capacity to set up and manage systems, training, and evaluation related to digital health within these settings as well as a lack of process and interoperability of these systems. These issues related to capacity has been reported previously in surveys, where senior clinicians have indicated that increased administrative burden, understaffing and increased pressure to meet regulatory demands are large contributors to work‐related burnout.^33^, ^34^ The lack of standardisation poses a significant challenge to the feasibility and scalability of digital health systems within healthcare settings. Without a unified, standardised and interoperable framework, the integration of digital health systems and devices into healthcare is unlikely to deliver the anticipated benefits.^35^

There were two strategies that were complementary to a digital health navigator in our study under the determinant ‘administration support’; both rated highly important but only one rated highly feasible. It was considered feasible when existing administrative staff were to perform this role rather than having a newly created position. However, there is value in having this role newly created as it could extend beyond administrative duties. This may also mitigate staff burnout as outlined above. A mix of review studies have indicated that a digital navigator could facilitate training, conduct evaluations of evidence‐based apps, enhance engagement, and liaise between technical and clinical teams.^36^, ^37^, ^38^, ^39^, ^40^, ^41^ In a nutrition‐specific context, for example, a digital navigator could assist with the verification process of digital food records, as this process can result in significant participant and software‐related error.^42^, ^43^ To our knowledge, there has been no literature to explore the digital navigator role in a nutrition‐specific context and warrants further research and/or feasibility testing to develop a business case for this role in outpatient settings.

Adhering to quality care was another key area that was rated highly important and feasible, such as auditing and evaluating digital health services including consumer‐reported outcome and experience measures. Healthcare providers consider it important to manage safety risks, as well as tailoring individual preferences and collecting data on effectiveness to improve services.^12^ Furthermore, one factor that could make healthcare providers view it as feasible is that digital health itself can be used to aid with the collection of these quality measures. A systematic review reveals that clinician stakeholders perceive that electronic patient‐reported outcome measures (ePROMs), which are integrated into electronic medical records, are a key facilitator to their implementation within healthcare settings.^44^ Nevertheless, a systematic review highlights that there is a need for further patient collaborations to understand outcomes important to patients, specifically related to outpatient dietetic care.^45^ This suggests a need to also assess this within a digital health context, and this should be considered as the next step in strategic digital health audit and evaluation planning.

Strategies that were also rated ‘very high’ for both importance and feasibility focused on upskilling and support such as having access to shared digital resources from across disciplines and services. There is strong evidence to indicate that healthcare providers recognise the importance of interdisciplinary collaboration and creating spaces to achieve this.^46^ It may have been regarded as feasible by our participants as a result of existing electronic resource platforms set up by the state health departments^47^, ^48^ and external resources dedicated to digital health.^49^, ^50^ However, it appears that some of the collated and indexed resources related to digital health have not been updated for some time, thus they do not reflect the latest evidence within this area. Moreover, the global nutrition library ‘Practice‐based Evidence in Nutrition’ (PEN) provides some digital nutrition resource pathways for healthcare providers,^51^ yet they offer limited practical guides for healthcare providers with using digital health (e.g., a list of evidence‐based nutrition apps/websites to support the management of various chronic conditions). A recent initiative ‘Grow and Go toolbook’ has collated and appraised existing nutrition resources for children under 5 years into a national database.^52^ This consolidation of resources into a single national database may serve as an effective approach to ensuring resources remain current and easily accessible to all end users. Additionally, it may provide insight of where to allocate investments into newly created digital health resources.

Other strategies that were rated very high for both importance and feasibility focused on individualising patient care, including offering choice in the model of care they receive. Healthcare providers have indicated that hybrid models are important to adapt care based on individual preferences and needs^17^; however, very few clinical trials have tested the impact of patient choice in digital healthcare delivery. A recent randomised trial indicated patient choice in how to access a suite of digital nutrition services was acceptable and feasible.^53^ Furthermore, existing infrastructure and prior experience of delivering hybrid models of care may have contributed to the high feasibility rating for this strategy.^17^ It is important to consider that not all healthcare providers would consider hybrid models to be feasible, with varying constraints to digital health resources and funding across hospitals in Australia.^54^ Nevertheless, a large part of the investment into hybrid healthcare in Australia so far has been related to video and telephone consultations with limited focus on mobile apps and remote patient monitoring.^4^ A 2023 systematic review of cost‐effectiveness studies indicates that mobile health modalities for delivering nutrition care appear to be cost‐effective from a health service perspective.^55^ However, the majority of the included studies were within‐trials (randomised controlled trials), with the authors indicating the need for broader considerations for health system economic factors related to these models of care. Thus, further stakeholder engagement could be highly valuable to identify perspectives on operating costs associated with mobile nutrition health to support individualised hybrid care.

This study has indicated the strategies related to providing options for digital health upskilling and support; adhering to quality of care with effective evaluation processes and individualising patient care are of priority for healthcare providers of nutrition care. Each of the key themes within the review aligns with key national and international initiatives.^5^, ^56^ This implies that there is an understanding among these healthcare providers of what needs to happen to successfully adopt and implement digital health.

There are strengths and limitations that are worth noting in this study. The revised CFIR framework^28^ was used in this study to guide the selection of determinants and the development of strategies. However, we were not able to use the associated CFIR‐ERIC Matching tool^57^ for strategy development as a version that aligned with the revised CFIR framework was not available at the time of the preparatory phase of this study. Thus, we may have missed out on some evidence‐informed strategies that could have aligned with this tool. Moreover, recruitment of a variety of healthcare providers including doctors and nurses, helped to capture broad perspectives on digital nutrition care which was missing from our previous work.^17^ However, we did have minimal ‘other’ allied health representation (e.g., no occupational therapists, speech pathologists and physiotherapists) who could have provided additional insights from their experiences and our expert panel were largely from Queensland. While the inclusion of administrative staff could have provided further perspectives on strategies related to administration support. Additionally, generalisability of the findings should be cautioned as participants were recruited from one public health district within Australia. Further considerations for generalisation include infrastructure and connectivity challenges in rural and remote communities, and the diverse preferences of cultural and linguistic groups, including First Nations peoples, in digital health adoption.^58^, ^59^, ^60^ While this study captured health system perspectives, future implementation would benefit from involving vulnerable communities, recognising that a flexible approach may enable resources to be redirected to those requiring intensive face‐to‐face support.

This study presents a refined list of strategies for digital health implementation that have been expertly rated based on their importance and feasibility, within a nutrition specific context. A high portion of the strategies presented were considered important but not always feasible indicating a need for additional resources such as deployment of personnel to support implementation. Strategies that met consensus and were prioritised mostly related to adhering to quality of care with effective evaluation processes; providing options for digital health upskilling and support, and individualising patient care. Facilitating implementation of these strategies may include reviewing and updating current electronic resource platforms, identifying patient‐reported measures for digital nutrition outpatient care and conducting further stakeholder engagement to identify perceived costs associated with operating mobile health. This work has highlighted an understanding by team leaders and senior clinicians, as well as dietetic and allied health directors on what needs to happen to successfully adopt and implement digital health models of nutrition care.

## AUTHOR CONTRIBUTIONS

*Conceptualisation*: AB, JTK and KTC. *Methodology*: AB, IJH, JTK and KTC. *Analysis*: AB. *First draft of the manuscript*: AB. *Editing, revisions and final approval*: AB, IJH, JTK and KTC. All authors are in agreement with the manuscript and declare that the content has not been published elsewhere.

## FUNDING INFORMATION

AB was supported by an Australian Government Research Training Program stipend for a Doctor of Philosophy program. JK was supported by a Postdoctoral Fellowships from the National Heart Foundation of Australia (106081).

## CONFLICT OF INTEREST STATEMENT

The authors declare no conflicts of interest.

## ETHICS STATEMENT

Ethical approval was granted from the Human Research Ethics Committees for Metro South Hospital and Health Service (HREC/2023/QMS/94426) and ratified by The University of Queensland (2023/HE001282).

## Supporting information


**Data S1.** Supporting Information.

## Data Availability

The data that support the findings of this study are available on request from the corresponding author. The data are not publicly available due to privacy or ethical restrictions.
