# Supplementary material for: Translating digital health services for nutrition care management of chronic conditions in outpatient settings: A multi‐stakeholder e‐Delphi study
Source: Nutr Diet. 2025 Feb 17;82(2):231–43. doi: 10.1111/1747-0080.12927 (PMC11973619; doi:10.1111/1747-0080.12927)
Supplement: Supplementary file 1 — Data S1. Supporting Information. [file NDI-82-231-s001.docx]

**Supplementary file for Translating digital health services for nutrition care management into clinical practice: a multi-stakeholder e-Delphi study**

**Table S1: Mapped determinants and strategies to the CFIR framework and literature**

| **CFIR domain and construct** | **Determinants, Questions and Strategies** | **References that informed determinants and strategies** |
| --- | --- | --- |
| Inner setting domain- structural characteristics  Or  Implementation process- planning  Or  Implementation process- Teaming | Administration support for healthcare providers  **Determinant:** Some healthcare providers perceived that digital health may increase workload and reduce efficiency. However, having access to ongoing administrative support for data collection, organisation of appointments and resource distribution was thought to facilitate scale up and adoption.  **Questions:**   1. How important are each of these strategies related to *administrative support for healthcare providers using digital health services to deliver nutrition care in outpatient settings?*   How feasible are each of these strategies related to *administrative support for healthcare providers using digital health services to deliver nutrition care in outpatient settings?*  **Strategies:**   - Involve allied health assistants to assist with administrative tasks related to digital health - Create a new digital health administrator officer role/s who would oversee all digital health administrative tasks - Investigate how the booking systems can be adapted to better support digital health services - A clearer understanding and outline of the different organization and staffing roles related to digital health | Barnett et al. 2022^1^  Thomas et al. 2022^2^  Seljedlid et al 2021^3^  Varsi et al 2021^4^  Sterling et al 2021^5^  Bashi et al 2018^6^ |
| Inner setting domain- culture  Or  implementation process-engaging | Engagement of health care providers  **Determinant:** Initially, health care providers indicated their reluctancy to use digital health, reverting to in person when the option was allowed. However, the ongoing success of digital health is dependent on the attitude of the whole team.  **Questions:**   1. How important are each of these strategies related to *the value, beliefs and norms of healthcare providers using digital health services to deliver nutrition care in outpatient settings?* 2. How feasible are each of these strategies related to *the value, beliefs and norms of healthcare providers using digital health services to deliver nutrition care in outpatient settings?*   **Strategies:**   - Promote telehealth to staff as a hybrid model that enhance consumer centered care - Designate digital health champions who would be responsible for promoting digital health services in their departments/wards - Share past digital health projects and positive experiences – sharing success using the inter-site and interdisciplinary meetings - Encouraging healthcare providers (particularly team leaders and directors) to be involved in digital nutrition research projects | Barnett et al. 2022^1^  Thomas et al. 2022^2^  Seljedlid et al 2021^3^  Varsi et al 2021^4^ |
| Inner setting- relative priority | Perceived consumer demand  Determinant: There is a perception among some health care providers that there is limited consumer demand for current digital health services offered in the hospital and outpatient setting. However, there is a need to understand the evolving perspectives of consumers as result of greater exposure to technology in recent times.  **Questions:**   1. How important are each of these strategies related to the *perceived consumer demand from healthcare providers using digital health services for delivering nutrition care in outpatient settings?* 2. How feasible are each of these strategies related to the *perceived consumer demand from healthcare providers using digital health services for delivering nutrition care in outpatient settings?*   **Strategies:**   - Promote telehealth to staff as a hybrid model that is evidence-based and enhances consumer centered care - Set and evaluate KPIs for digital nutrition service attendance, bookings, and cancellations | Barnett et al. 2022^1^  Thomas et al. 2022^2^  Varsi et al 2015^4^ |
| Inner setting- mission alignment | Maintain quality of care  **Determinant:** It is important for healthcare providers to deliver the same high-quality care using digital health  **Questions:**   1. How important are each of these strategies for maintaining quality of care? 2. How feasible are each of these strategies for maintaining quality of care?   **Strategies:**   - Audit and evaluate digital health services including consumer reported outcome and experience measures - Establish ongoing quality improvement projects - Mandate reporting and mitigation of adverse events that occur during telehealth appointments | Barnett et al. 2022^1^  Thomas et al. 2022^2^ |
| Inner setting domain- access to knowledge and information | Training for health care providers  **Determinant:** Healthcare providers don’t always feel confident with digital health e.g Nutritional physical assessments. However, training has resulted in greater confidence with conducting digital health service delivery.  **Questions:**   1. How important are each of these related to *healthcare provider training in digital health to deliver nutrition care in outpatient settings?* 2. How feasible are each of these related to *healthcare provider training in digital health to deliver nutrition care in outpatient settings?*   **Strategies:**   - Conduct discipline specific assessments, rapport building and online communication, simulated practice sessions - Provide staff delivering services with ‘how to’ guides and troubleshooting guides - Include digital service training and skills in mandatory annual training | Barnett et al. 2022^1^  Thomas et al. 2022^2^ |
| Inner setting- access to knowledge and information | Training for patients  **Determinant:** Patients have varying levels of digital literacy, where some have confidence to use digital health services while others desire onboarding and technical support.  **Questions:**   1. How important are each of these strategies related to *training for patients who are using digital health to receive nutrition care within outpatient settings?* 2. How feasible are each of these strategies related to *training for patients who are using digital health to receive nutrition care within outpatient settings?*   **Strategies**   - Provide consumers with ‘how to’ guides and troubleshooting guides - Have onboarding and technical support available for those who need | Barnett et al. 2024 ^7^  Gibson et al. 2020^8^ |
| Inner setting domain- communications | Availability of digital resources and learnings  **Determinant:** Sharing digital health resources is important to health care providers but they are uncertain where to find them.  **Questions**   1. How important are each of these strategies related to the *sharing of digital health resources and learnings for healthcare providers using digital health services to deliver nutrition care in outpatient settings?* 2. How feasible are each of these strategies related to the *sharing of digital health resources and learnings for healthcare providers using digital health services to deliver nutrition care in outpatient settings?*   **Strategies:**   - Access to a library of digital resources to enable consolidation of resources across disciplines, services, and professional bodies. - Use existing meetings to monitor implementation of digital health interventions | Barnett et al. 2022^1^  Thomas et al. 2022^2^  Seljedlid et al 2021^3^ |
| Characteristic – need/capability | Dietary information that is individualised  **Determinant:** There is a strong desire to received individualised care in digital health with some evidence suggesting it can improve adherence.  **Proposed Question:**   1. How important are each of these strategies related to *individualising* *dietary information delivered by digital health services in outpatient settings?* 2. How feasible are each of these strategies related to *individualising* *dietary information delivered by digital health services in outpatient settings?*   **Strategies:**   - Continue to provide ongoing telehealth or face to face in clinic appointments with dietitians for patients to receive individualised information when using mobile or web- based services. - Dietitians need to individualise the material that is shared to patients through digital health. - Allow for mobile and web-based services to have a feature where it is customizable, and the settings can be changed to suit the patient/users e.g number of push notifications received. | Barnett et al. 2022^2^  Barnett et al. 2024^7^  Dawson et al. 2021^9^  Donald et al. 2022^10^  Kelly et al. 2019 ^11^ |

1. Barnett A, Kelly JT, Wright C, Campbell KL. Technology-supported models of nutrition care: Perspectives of health service providers. Digit Health 2022;8:20552076221104670.

2. Thomas EE, Taylor ML, Ward EC, Hwang R, Cook R, Ross JA, et al. Beyond forced telehealth adoption: A framework to sustain telehealth among allied health services. J Telemed Telecare 2022:1357633x221074499.

3. Seljelid B, Varsi C, Solberg Nes L, Øystese KA, Børøsund E. A Digital Patient-Provider Communication Intervention (InvolveMe): Qualitative Study on the Implementation Preparation Based on Identified Facilitators and Barriers. J Med Internet Res 2021;23(4):e22399.

4. Varsi C, Stenehjem AE, Børøsund E, Solberg Nes L. Video as an alternative to in-person consultations in outpatient renal transplant recipient follow-up: a qualitative study. BMC Nephrol 2021;22(1):105.

5. Sterling WA, Sobolev M, Van Meter A, Guinart D, Birnbaum ML, Rubio JM, et al. Digital Technology in Psychiatry: Survey Study of Clinicians. JMIR Form Res 2022;6(11):e33676.

6. Bashi N, Hassanzadeh H, Varnfield M, Wee Y, Walters D, Karunanithi M. Multidisciplinary Smartphone-Based Interventions to Empower Patients With Acute Coronary Syndromes: Qualitative Study on Health Care Providers' Perspectives. JMIR Cardio 2018;2(2):e10183.

7. Barnett A, Catapan SC, Jegatheesan DK, Conley MM, Keating SE, Mayr HL, et al. Patients' acceptability of self-selected digital health services to support diet and exercise among people with complex chronic conditions: Mixed methods study. Digit Health 2024;10:20552076241245278.

8. Gibson CA, Gupta A, Greene JL, Lee J, Mount RR, Sullivan DK. Feasibility and acceptability of a televideo physical activity and nutrition program for recent kidney transplant recipients. Pilot and Feasibility Studies 2020;6(1):126.

9. Dawson J, Campbell KL, Craig JC, Tong A, Teixeira-Pinto A, Brown MA, et al. A Text Messaging Intervention for Dietary Behaviors for People Receiving Maintenance Hemodialysis: A Feasibility Study of KIDNEYTEXT. Am J Kidney Dis 2021;78(1):85-95.e1.

10. Donald M, Beanlands H, Straus S, Smekal M, Gil S, Elliott MJ, et al. An eHealth self-management intervention for adults with chronic kidney disease, My Kidneys My Health: a mixed-methods study. CMAJ Open 2022;10(3):E746-e54.

11. Kelly JT, Warner MM, Conley M, Reidlinger DP, Hoffmann T, Craig J, et al. Feasibility and acceptability of telehealth coaching to promote healthy eating in chronic kidney disease: a mixed-methods process evaluation. BMJ Open 2019;9(1):e024551.

| **Table S2: Full results for the Final Prioritisation Survey** | | | |
| --- | --- | --- | --- |
| Domain | Strategy | Top priority | Frequency |
| Administration | Provide training to administrative officers to perform a digital health administrative role. | #1 | 8 |
|  | Develop a business case to support creation of new digital health administrator roles. | #2 | 6 |
|  | Investigate how the booking systems can be adapted to better support digital health services. | #3 | 4 |
| Value | Promote telehealth to staff as a hybrid model that can enhance consumer centered care. | #1 | 10 |
|  | Designate digital health champions who would be responsible for promoting digital health services in their departments/wards. | #2 | 4 |
|  | Encouraging healthcare providers (particularly team leaders and directors) to be involved in digital nutrition research projects | #2 | 4 |
| Consumer | Promote telehealth to staff as a hybrid model that is evidence-based and enhances consumer centered care. | #1 | 7 |
|  | Set and evaluate KPIs for digital nutrition services which are realistic for the health care provider, considers the consumer demand and consumer suitability for the modality of contact. | #2 | 6 |
|  | Educating health care providers of the perceived acceptability of digital health services across all groups. | #3 | 5 |
| Quality | Audit and evaluate digital health services including consumer reported outcome and experience measures. | #1 | 14 |
|  | Establish ongoing quality improvement projects. | #2 | 3 |
|  | Mandate reporting and mitigation of adverse events that occur during telehealth appointments. | #3 | 1 |
| Training | Provide staff delivering services with ‘how to’ guides and troubleshooting guides. | #1 | 7 |
|  | Conduct discipline specific assessments, rapport building and online communication, simulated practice sessions. | #2 | 6 |
|  | Provide staff the option to participate in training related to digital health services. | #3 | 5 |
| Patient support | Provide consumers with ‘how to’ guides and troubleshooting guides. | #1 | 8 |
|  | Have onboarding and technical support available for those who need and make this available over the weekend and after work hours. | #2 | 7 |
|  | Develop a business case for consumers to receive after hours technical support. | #3 | 3 |
| Resource | Access to a library of digital resources to enable consolidation of resources across disciplines, services, and professional bodies. | #1 | 14 |
|  | se existing meetings to monitor implementation of digital health interventions. | #2 | 4 |
| Individual | Continue to provide ongoing appointments (in-person or video telehealth) with dietitians, to those who request.  And  Allow for mobile and web-based services to have a feature where it is customisable, and the settings can be changed to suit the patient/users e.g. number of push notifications received. | #1 | 8 &8 |
|  | Dietitians need to individualise the material that is shared to patients through digital health. | #2 | 2 |
